# Obesity in cystic fibrosis

**DOI:** 10.1016/j.jcte.2021.100276

**Published:** 2021-11-17

**Authors:** Katherine A. Kutney, Zahrae Sandouk, Marisa Desimone, Amir Moheet

**Affiliations:** a Department of Pediatrics, Division of Pediatric Endocrinology, University Hospitals, Rainbow Babies and Children’s Hospital Case Western Reserve University, 11100 Euclid Ave, Suite 737, Cleveland, OH 44106, USA; b Department of Internal Medicine, Division of Endocrinology, University of Michigan, USA; c Division of Endocrinology, Diabetes, and Metabolism, SUNY Upstate Medical University, USA; d Department of Medicine, University of Minnesota, USA

**Keywords:** CF, cystic fibrosis, CFRD, cystic fibrosis related diabetes, BMI, body mass index, Cystic fibrosis, Obesity, Cystic fibrosis related diabetes, Cardiovascular disease, CFTR modulator

## Abstract

The prevalence of obesity in patients with cystic fibrosis (CF) is increasing and around one-third of adults with CF are now overweight or obese. The causes of excess weight gain in CF are likely multifactorial, including: adherence to the high-fat legacy diet, reduced exercise tolerance, therapeutic advances, and general population trends. Increased weight has generally been considered favorable in CF, correlating with improved pulmonary function and survival. While the optimal BMI for overall health in CF is unknown, most studies demonstrate minimal improvement in pulmonary function when BMI exceeds 30 kg/m^2^. Dyslipidemia and cardiovascular disease are important co-morbidities of obesity in the general population, but are uncommon in CF. In people with CF, obesity is associated with hypertension and higher cholesterol levels. With longer life expectancy and rising obesity rates, there may be an increase in cardiovascular disease among people with CF in coming years. Overweight CF patients are more likely to be insulin resistant, taking on features of type 2 diabetes. Treating obesity in people with CF requires carefully weighing the metabolic risks of overnutrition with the impact of low or falling BMI on lung function. This article describes current knowledge on the epidemiology, causes, consequence, and treatment of obesity in people with CF.

## Introduction

Cystic fibrosis (CF) is an autosomal recessive, multi-system disorder characterized by progressive pulmonary disease and premature mortality. CF has classically been associated with a negative energy balance, malnutrition and increased energy expenditure related to chronic inflammation and frequent pulmonary infections [1]. A high calorie, high fat diet has been the standard of care for several decades, because nutritional status is an important predictor of pulmonary function and survival in people with CF [2], [3]. With an epidemic of obesity overtaking the general population, there has also been a surprising increase in overweight and obesity in the CF population [4], [5]. The impact of overweight and obesity on CF outcomes such as pulmonary function, diabetes and cardiometabolic risk warrants further investigation, especially as this population continues to have improvements in life expectancy.

## Epidemiology

Studies of overweight and obesity in people with CF demonstrate a rising prevalence of over time. Overweight is defined as body mass index (BMI) 25–29.9 kg/m^2^ for adults or 85–94.9th percentile for youth; while obesity is defined as BMI ≥ 30 kg/m^2^ for adults and ≥ 95th percentile for youth. These standard definitions for overweight and obesity are used to describe people with CF, unless otherwise noted [6]. In 2005, Kastner-Cole reported the prevalence of overweight and obesity in United Kingdom patients with CF due to homozygous mutation of ΔF508 (CFΔF508) to be 9.1% and 1.1%, respectively [7]. Stephenson evaluated longitudinal changes in nutrition at the Toronto CF Center, comparing the adult nutritional status from before 1990 to after 2000. Underweight decreased from 20.6% to 11.1% while overweight/obese increased from 7% to 18.4% over this time period [8]. More recently, Harindhanuvadi evaluated 484 adults at the University of Minnesota CF Center and found 25.6% of patients were overweight and 6.6% were obese [9].

Rates of overweight and obesity may also vary by country. Single center studies performed between 2007 and 2012 in the US, Greece, and Spain report combined rates of overweight and obesity of 23%, 13.2%, and 7%, respectively [10], [11], [12]. With growing recognition of overnutrition in CF, national registries have begun reporting overnutrition statistics. According to the 2019 US CF Foundation registry report 23.1% of adults with are overweight and 8.3% are obese and the prevalence of adult overweight/obesity has more than doubled since 1999, when it was 12.8% [5]. The Australian CF Registry report defines overnutrition differently, deeming BMI > 27 kg/m^2^ “high BMI.” In 2017, 17% of Australian adults with CF had high BMI and 11.2% of youth ages 2–18 were overweight (BMI 85–94.9%) or obese (BMI ≥ 95%) [4]. The European Cystic Fibrosis Society 2018 annual report provides the maximum BMI among adults with CF for each European country. While most larger countries include obese individuals with CF, it is noteworthy that eight of 31 countries report at least one patient with BMI > 40 kg/m^2^, highlighting that extreme obesity can occur in individuals with CF [13].

Preventing obesity in people with CF requires understanding risk factors for excess weight gain. Pancreatic insufficiency impacts approximately 85% of people with CF and has long been associated with malnutrition, so may also result in lower risk for obesity [5]. In fact, multiple studies find higher rates of exocrine pancreatic sufficiency and mild genotypes among overweight and obese cohorts [8], [9], [11], [14], [15]. Nevertheless, pancreatic insufficient individuals with CF are not immune to overnutrition. The Australian registry report found that 18% of adults with pancreatic insufficiency experienced “high BMI” compared to 30% of those with pancreatic sufficiency [4]. A number of studies have found higher rates of overweight and obesity among males [7], [8], [9], [16], while other studies found no sex differences [12], [14]. Increasing age is associated with rising BMI among the general population and has been found to predict obesity in some CF studies as well [8], [9], [14]. Interestingly this was not found in the Italian cohort, suggesting cultural factors may influence obesity risk factors across the globe [15].

## Causes of overweight and obesity in people with CF

### Dietary recommendations

People with CF are generally advised to consume a high fat, high calorie diet to overcome the increased metabolic rate and malabsorption associated with CF (Table 1). This recommendation stems from the observed clinical improvement in the US CF population after implementing the high-fat, high-calorie diet previously recommended at the Toronto CF center [17]. Unfortunately, this intense focus on a high-fat, high calorie diet has resulted in poor diet quality for many people living with CF. Nutritional surveys from Australia and Europe describe excessive intake of energy dense, nutrient poor foods, simple sugar and saturated fats [18], [19]. Childhood eating habits strongly influence adult eating behaviors in general population studies, and it is likely that people with CF will have difficulty transitioning to a lower-calorie diet after a lifetime of consuming high-calorie, high-fat foods [20].

**Table 1:** Summary of Nutrition Guidance by Country. RDA = recommended daily allowance. BMI = body mass index.

Country	Year	Nutrition Advice	BMI target	Overnutrition
Australia [50]	2017	110–200% RDA calories Adequate fat to meet calorie needs.	Youth: 50–<85%le Adult female: 22–27 kg/m^2^ Adult male: 23–27 kg/m^2^	Children > 85%le Adults > 27 kg/m^2^
US [3], [36]	2002, 2008	110–200% RDA calories 30–40% calories from fat	Youth: >50%le, Adult female: >22 kg/m^2^ Adult Male: >23 kg/m^2^	Not defined
Europe [55]	2016	110–200% RDA calories adequate fat to prevent weight loss and promote muscle; focus on adequate protein	Youth 2–18: BMI 50%le Adult female: >22 kg/m^2^ Adult Male: >23 kg/m^2^	Not defined

### Physical activity restriction:

Physical activity is an important component of a healthy lifestyle and may help with maintenance of a healthy weight [21]. Among the general population, engaging in regular exercise is associated with having a healthy BMI, as well as increased lean muscle mass and lower risk for cardiometabolic complications [22]. Engaging in regular exercise is also an important component of CF therapy that may slow the decline in pulmonary function [23]. Physical activity levels vary greatly among people with CF, and pulmonary symptoms during exercise may serve as a deterrent to engaging in regular physical activity [24], [25]. Furthermore, patients with severe pulmonary disease are less likely to engage in physical activity and may require supplemental oxygen during exercise [26], [27]. Although, physical activity has not been directly linked to overweight or obesity in people with CF, perceived exercise intolerance may contribute to physical inactivity and excess weight gain in people with CF.

### Modulators

CFTR modulators are a revolutionary treatment advance, partially restoring function to the dysfunctional, disease-causing CFTR channel, and are, therefore, likely to ameliorate undernutrition in CF. In fact, many studies have demonstrated weight gain in modulator-treated patients [28], [29]. A recent systematic review examining 13 CFTR modulator trials performed between 2012 and 2017 found strong evidence for weight gain in ivacaftor-treated patients with a G511D mutation, but weaker evidence for weight gain with liumacaftor/tvacaftor or tezacaftor/ivacaftor treatment [29]. Elexacaftor/tezacaftor/ivacaftor (ETI), approved in late 2019, was not included in the systematic review. ETI is a “highly effective” CFTR modulator for individuals with eligible CFTR variants, providing similar level of CFTR function as ivacaftor. A 5-year study of the US and UK registries demonstrated BMI increase of 2.4 kg/m^2^ and 1.9 kg/m^2^ respectively in ivacaftor-treated cohorts [28]. While longitudinal data for ETI is not available, a single-center study of 94 adults found an average of 2.4% increase in weight after 3 or more months of ETI therapy. Importantly, patients previously treated with a CFTR modulator experienced less weight gain [30].

The mechanisms of weight gain with CFTR modulator therapies have been best studied for ivacaftor. Stallings et al. evaluated 23 youth and adults with CFTR gating mutations before and 3 months after initiation of ivacaftor. Ivacaftor-treated subject demonstrated improved fat absorption, reduced gut inflammation, and reduced resting energy expenditure. Fecal elastase, a measure of exocrine pancreatic function, improved only in the pancreatic sufficient subjects [31]. Similar physiologic changes are likely to occur with ETI therapy, as both are highly effective in restoring CFTR function. Improvement in gastrointestinal discomfort may also contribute to weight gain after modulator therapy. A study of 102 adults with CF found improvement in patient’s self-perception of eating, body image, and weight after ivacaftor treatment [32]. Lastly, CFTR modulators must be taken with a high-fat meal, which may increase fat intake and promote weight gain.

### General population trends

**Fig. 1:**
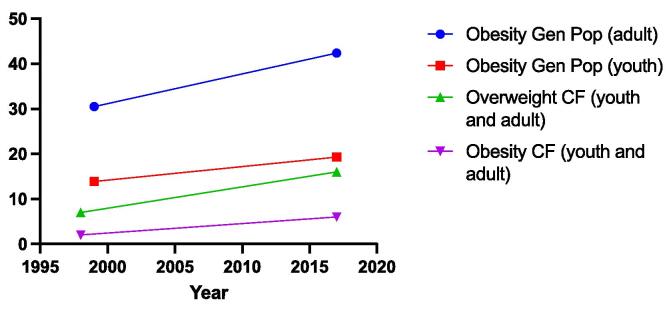
Overweight and obesity trends for people with CF and the general population. The percent of the general population meeting criteria for obesity, separated by youth (red square) and adult (blue circle) between 1999 and 2018. (Source: NHANES) The percent of the CF population (youth and adult) meeting criteria for overweight (green upright triangle) and obesity (purple downfacing triangle) between 1997 and 2018. (Source: Flume, NACFC, 2019). Adult overweight is BMI 25–29.9 kg/m^2^, adult obesity is BMI ≥ 30 kg/m^2^, youth overweight is BMI 85–94.9%, youth obesity is BMI ≥ 95%. Gen Pop = general population.

Rates of overweight and obesity among the general population have risen dramatically over the past 40 years. In the United States, just 5.5% of children and 15% of adults were obese in 1980; while, in 2018 19.3% of children and 42.8% of adults were obese [33], [34]. Similar rates of increase have been seen among people with CF (Fig. 1). While the causes of the obesity epidemic are incompletely understood, factors related to the modern lifestyle including consumption of calorie-dense foods and low levels of physical activity are widely implicated [35]. Rising BMI among individuals with CF over the past four decades primarily reflect therapeutic advances and intense nutritional counseling, but may be partially driven by societal trends [36]. As people with CF become more physiologically similar to the general population with the advent of CFTR modulator therapies, the factors driving obesity in the general population may become increasingly relevant to people with CF.

## Clinical consequences of obesity in CF

### Pulmonary function

Traditionally, it has been well established that weight loss and malnutrition in patients with CF are associated with worse prognosis; the lower the BMI (body mass index) the lower the lung function [5]. As overweight and obesity increase among people with CF, it is important to understand if this relationship has a limit. Specifically, it is imperative to determine whether there an upper BMI cut-off after which pulmonary function no longer improves or even begins to decline. Kasner-Cole studied youth and adults with homozygous F508del variants and found a positive association between FEV1 and BMI up to 23 kg/m^2^ in adults and across all BMI z-scores in youth [7]. Stephenson, reported a threshold of BMI of 25 kg/m^2^, above which lung function improvement with weight gain becomes minimal in adults with CF [8]. In this study they also reported that the greatest lung benefit was seen with increase in BMI in the undernourished CF patients as opposed to overweight CF patients [8]. Harindhanuvadhi found higher FEV1 and fewer pulmonary exacerbations in overweight or obese adults with CF, though improvement in pulmonary function was minimal above a BMI of 28–29 kg/m^2^ [9]. Obesity is associated with reduced lung volume, forced vital capacity, and FEV1 in the general population, so may be expected to negatively impact pulmonary function among individuals with CF [37]. Interestingly, a study of adults with CF found that higher adiposity was associated with lower lung function, even in individuals with normal weight obesity [38]. For more information about body composition measures in people with CF, please see the article by Soltman and colleagues in this issue.

### Sleep disordered breathing

Another known complication of obesity in the general population is sleep disordered breathing such as obstructive sleep apnea. Studies have reported a prevalence of sleep apnea as high as 57% in children with CF and an association with low BMI [39]. Interestingly, another study found that rates of sleep disordered breathing were similar between adult patients with and without CF [40]. A more recent study, showed a three times higher odds of moderate to severe sleep disordered breathing in children and adult patients with CF compared to their non CF counterparts. Interestingly, the frequency was not higher in those with higher BMI (>25 in adults or > 85th percentile in children) [41]. Given the strong association between obesity and sleep apnea in the general population, future studies in obese individuals with CF should be performed.

### Transplant candidacy

Obesity not only affects lung function itself but can also impact management of CF. BMI has become part of the recently proposed BODE index (BMI, airflow obstruction, degree of dyspnea and exercise capacity) for the measurement of health-related quality of life and candidacy for transplant. The index increases as BMI decreases; thus, a higher BMI may impact transplant candidacy. In fact, according to the international guidelines for the selection of lung transplant candidates, a BMI above 30 kg/m^2^ is now a relative contraindication to transplant [42]. Transplant candidacy should be considered when weighing the potential risks and benefits of therapeutic weight loss in obese people with CF.

### Insulin resistance and diabetes

Cystic fibrosis related diabetes (CFRD) affects approximately 20% of youth and 50% of adults with CF, and it is estimated that > 80% of pancreatic insufficient individuals develop CFRD with increasing age [43]. The pathophysiology of CFRD involves both insulin insufficiency and insulin resistance, combining features of type 1 and type 2 diabetes. Furthermore, CFRD progresses along a spectrum with patients gradually progressing from normal glucose tolerance, to impaired glucose tolerance, then to CFRD over time [43]. Earlier studies suggested that progressive insulin deficiency was the primary driver of CFRD progression [44]. As people with CF become overweight and obese, insulin resistance may play a larger role in CFRD pathogenesis. A study of 290 adults with CF in Montreal demonstrated an association between high BMI, defined as > 27 kg/m^2^ and insulin resistance on a frequently sampled OGTT [14]. Insulin resistance is also associated with increasing age, a fact that may become more important as the CF population ages. A longitudinal study of 49 adults with CF over age 35 years found that increasing insulin resistance, not progressive insulin deficiency, was the primary driver of CFRD progression in that cohort [45]. Whether people with CFRD and insulin resistance should be labeled as having type 2 diabetes is an area of ongoing debate, with important implications for diabetes treatment. At this time, guidelines only recommend insulin treatment for CFRD [46].

### Cardiovascular disease/dyslipidemia

Obesity is associated with worsened cardiovascular outcomes as well as metabolic profile in the general population, but the data on this remains limited in the CF population. A single center study looking at the prevalence and pulmonary/cardiovascular risk factors associated with overweight and obesity in CF patients showed that the prevalence of hypertension was higher in overweight (25%) and obese (31%) than normal (17%) or underweight (16%) individuals, p = 0.01. Total cholesterol levels were higher in overweight/obese versus normal/underweight (144–147 vs 123–131 mg/dL, p = 0.04) as were LDL levels (70–71 vs 53–60 mg/dL, p = 0.02), but all were within the normal range [9]. The single-center study by Bonhore also demonstrated an association between over-nutrition and higher blood pressure and LDL-cholesterol, as well as insulin resistance [14]. The risk of hepatic steatosis may also be higher in overweight patients with CF (BMI > 25 kg/m^2^). A retrospective study of 114 adults found that hepatic steatosis was associated with higher BMI and lung function, but there was no significant association with pancreatic insufficiency or CFRD [47]. With rapidly expanding access to CFTR modulators, longer life-spans, and increasing obesity, people with CF may be at risk of serious cardiovascular complications in the near future. More research is needed to understand the impact of CFTR modulators on nutritional status, body composition and its consequences for cardio-metabolic health.

## Treatment of obesity in CF

Data regarding the treatment of obesity in CF is extremely limited, but extrapolation from general population guidelines provides some insight. Dietary change is generally the first treatment recommend for obesity, and caloric reduction of 500–1000 kcal/day is often sufficient to produce 1-2lbs per week of weight loss. No specific diet has been proven to be superior to any other, though low carbohydrate diets have garnered attention recently [48]. The Endocrine Society recommends pharmacologic therapy for adults with BMI > 27 kg/m^2^ with a comorbidity or > 30 kg/m^2^ without comorbidity [49]. The threshold for implementing bariatric surgery is somewhat higher with BMI > 35 kg/m^2^ with a comorbidity or > 40 kg/m^2^ without comorbidity [48].

Weight-loss therapy in people with CF is complicated by the need to balance the risk of declining BMI on lung function with the possible metabolic advantages of lower BMI. The Australia and New Zealand CF Nutrition guidelines are the first to address the issue of overnutrition in CF, recommending a “high BMI” threshold of > 27 kg/m^2^ for CF (Table 1) [50]. The authors recommend reducing energy intake and increasing physical activity as the primary weight-loss interventions, extrapolating from general population evidence. While there is no evidence to support a specific weight-loss approach for people with CF, experts generally support consumption of nutrient-rich foods and avoidance of highly processed food [51]. Given the potential risks of reduced pulmonary function or loss of lean body-mass, overly restrictive diets, like a low-carb diet, carry additional risks in people with CF.

Medical therapy for obesity could be considered in select individuals with cystic fibrosis. Among the general population, multiple medical therapies are FDA approved to treat weight loss, but adverse effect profiles limit widespread use [49] (Table 2). Specific considerations exist for the use of weight loss medications in CF. Orlistat works by inhibiting pancreatic lipase and preventing fat absorption, key features of GI pathology in cystic fibrosis. Due to increased risk for fat soluble vitamin deficiencies, Orlistat use in people with CF is inadvisable [49]. GLP-1 receptor agonists should be avoided in pancreatic sufficient individuals with CF due to increased risk for pancreatitis [52].

**Table 2:** Pharmacologic treatments for obesity.

Medication Class	Generic name	Weight Loss	Side Effect	CF Specific Concern
Sympathomimetic amines [56]	Phentermine, diethylproprion	3–3.6 kg over 2–52 weeks	Adrenergic (tachycardia, hypertension, nervousness)	
Sympathomimetic amine and Antiepileptic [56]	Phentermine- topiramate	6.6–8.8 kg extra weight over 56 weeks	Adrenergic (tachycardia, hypertension, nervousness); concentration problems	
Lipase inhibitor [57]	Orlistat	3 kg additional weight loss over 1–4 years	Flatulence, oily stools; vitamin deficiencies	Fat soluble vitamin deficiency
Domamine/Norepinephrine reuptake inhibitor [58]	Buproprion/Naltrexone	3–5% weight loss after 56 weeks	Headache, nausea. Hepatotoxicity has been reported	Lumacaftor may alter Buproprion metabolism
GLP-1 Receptor Agonists [59], [60]	Liraglutide, Semaglutide	5.4 kg over 52 weeks; 12.4 kg over 36 weeks	Nausea, vomiting diarrhea. Pancreatitis.	Pancreatitis limits use in exocrine pancreatic sufficient patients, GI side effects, gastroparesis

Gastric bypass surgery is more effective in producing long-term weight loss than medical therapies [53]. Roux-en-y gastric bypass involves reducing the stomach size and removing small bowl from continuity, inducing malabsorption. Sleeve gastrectomy and gastric band procedures restrict the size of the stomach without altering intestinal continuity [54]. Gastric bypass surgery carries risk for vitamin and mineral deficiencies, including vitamin D deficiency. Malabsorption and vitamin deficiencies are a key feature of CF and may be exacerbated by metabolic surgery. Therefore, gastric bypass surgery should be used with extreme caution in individuals with CF after careful risk-benefit analysis.

## Conclusions

Life expectancy and overall health for people living with CF have improved dramatically over the last several decades. Much of this progress is built upon intense nutritional therapy, with early prescription of a high-fat, high-calorie diet and pancreatic enzyme supplementation. CFTR modulator therapies have accelerated the health of people living with CF, providing the opportunity to lead longer, healthier and more typical lives. Overnutrition is a rapidly growing problem among people with CF, mirroring the obesity epidemic in the general population. The degree to which obesity complications like insulin resistance, dyslipidemia and cardiovascular disease will impact people with CF remains uncertain. Furthermore, a new obese phenotype of CF related diabetes is emerging and little data exists to support optimal management. In face of this uncertainty, careful risk benefit analysis is needed to determine the most appropriate therapeutic prescriptions for people living with CF to ensure a healthy future.

## CRediT authorship contribution statement

KK, ZS, and MD contributed to writing of the original draft. KK and AM contributed to writing - reviewing & editing. AM provided supervision of this project.

## Funding

KK, ZS, MD and AM received support from the Cystic Fibrosis Foundation EnVision 2: Emerging Leaders in CF Endocrinology Program.

## Declaration of Competing Interest

The authors declare that they have no known competing financial interests or personal relationships that could have appeared to influence the work reported in this paper.

## References

[1] Culhane S., George C., Pearo B., Spoede E. (2013). Malnutrition in cystic fibrosis: A review. Nutr Clin Pract.

[2] Yankaskas J.R., Marshall B.C., Sufian B., Simon R.H., Rodman D. (2004). Cystic Fibrosis Adult Care: Consensus Conference Report. Chest.

[3] Borowitz D., Baker R.D., Stallings V. (2002). Consensus report on nutrition for pediatric patients with cystic fibrosis. J Pediatr Gastroenterol Nutr.

[4] Ahern S., Sims G., Tacey M., Esler M., Oldroyd J., Dean J. (2017). Australian Cystic Fibrosis Data Registry Annual Report.

[5] Cystic Fibrosis Foundation Patient Registry. 2019 Annual Data Report 2019.

[6] Centers for Disease Control and Prevention: Overweight and Obesity 2021. https://www.cdc.gov/obesity/data/index.html (accessed July 27, 2021).

[7] Kastner-Cole D., Palmer C.N.A., Ogston S.A., Mehta A., Mukhopadhyay S. (2005). Overweight and obesity in ΔF508 homozygous cystic fibrosis. J Pediatr.

[8] Stephenson A.L., Mannik L.A., Walsh S., Brotherwood M., Robert R., Darling P.B. (2013). Longitudinal trends in nutritional status and the relation between lung function and BMI in cystic fibrosis: A population-based cohort study. Am J Clin Nutr.

[9] Harindhanavudhi T., Wang Q.i., Dunitz J., Moran A., Moheet A. (2020). Prevalence and factors associated with overweight and obesity in adults with cystic fibrosis: A single-center analysis. J Cyst Fibros.

[10] Hanna R.M., Weiner D.J. (2015). Overweight and obesity in patients with cystic fibrosis: A center-based analysis. Pediatr Pulmonol.

[11] Panagopoulou P., Fotoulaki M., Nikolaou A., Nousia-Arvanitakis S. (2014). Prevalence of malnutrition and obesity among cystic fibrosis patients. Pediatr Int.

[12] González Jiménez D., Muñoz-Codoceo R., Garriga-García M., Molina-Arias M., Álvarez-Beltrán M., García-Romero R. (2017). Excess weight in patients with cystic fibrosis: is it always beneficial?. Nutr Hosp.

[13] Zolin A, McKone E, van Rens J. ECFS Patient Registry Annual Data Report 2018; 2018.

[14] Bonhoure A., Boudreau V., Litvin M., Colomba J., Bergeron C., Mailhot M. (2020). Overweight, obesity and significant weight gain in adult patients with cystic fibrosis association with lung function and cardiometabolic risk factors. Clin Nutr.

[15] Gramegna A., Aliberti S., Contarini M., Savi D., Sotgiu G., Majo F. (2021). Overweight and obesity in adults with cystic fibrosis: An Italian multicenter cohort study. J Cyst Fibros.

[16] Gramegna A., Contarini M., Aliberti S., Casciaro R., Blasi F., Castellani C. (2020). From ivacaftor to triple combination: A systematic review of efficacy and safety of cftr modulators in people with cystic fibrosis. Int J Mol Sci.

[17] Lai H.C., Corey M., Fitzsimmons S., Kosorok M.R., Farrell P.M. (1999). Comparison of growth status of patients with cystic fibrosis between the United States and Canada. Am J Clin Nutr.

[18] Calvo-Lerma J., Hulst J., Boon M., Martins T., Ruperto M., Colombo C. (2019). The Relative Contribution of Food Groups to Macronutrient Intake in Children with Cystic Fibrosis: A European Multicenter Assessment. J Acad Nutr Diet.

[19] Sutherland R., Katz T., Liu V., Quintano J., Brunner R., Tong C.W. (2018). Dietary intake of energy-dense, nutrient-poor and nutrient-dense food sources in children with cystic fibrosis. J Cyst Fibros.

[20] Kvaavik E., Lien N., Tell G.S., Klepp K.I. (2005). Psychosocial predictors of eating habits among adults in their mid-30s: The Oslo youth study follow-up 1991–1999. Int J Behav Nutr Phys Act.

[21] Hankinson A.L., Daviglus M.L., Bouchard C., Carnethon M., Lewis C.E., Schreiner P.J. (2010). Maintaining a high physical activity level over 20 years and weight gain. J Am Med Assoc.

[22] Kodama S., Saito K., Tanaka S., Maki M., Yachi Y., Asumi M. (2009). Cardiorespiratory Fitness as a Quantitative Predictor of All-Cause Mortality and Cardiovascular Events. J Am Med Assoc.

[23] Schneiderman J.E., Wilkes D.L., Atenafu E.G., Nguyen T., Wells G.D., Alarie N. (2014). Longitudinal relationship between physical activity and lung health in patients with cystic fibrosis. Eur Respir J.

[24] Swisher PT A.K., PT M.E. (2008). Perceptions of physical activity in a group of adolescents with cystic fibrosis. Cardiopulm Phys Ther J.

[25] Savi D., Quattrucci S., Internullo M., De Biase R.V., Calverley P.M.A., Palange P. (2013). Measuring habitual physical activity in adults with cystic fibrosis. Respir Med.

[26] Savi D., Simmonds N., Di Paolo M., Quattrucci S., Palange P., Banya W. (2015). Relationship between pulmonary exacerbations and daily physical activity in adults with cystic fibrosis. BMC Pulm Med.

[27] Bradley S., Solin P., Wilson J., Johns D., Walters E.H., Naughton M.T. (1999). Hypoxemia and hypercapnia during exercise and sleep in patients with cystic fibrosis. Chest.

[28] Volkova N., Moy K., Evans J., Campbell D., Tian S., Simard C. (2020). Disease progression in patients with cystic fibrosis treated with ivacaftor: Data from national US and UK registries. J Cyst Fibros.

[29] Bailey J., Rozga M., McDonald C.M., Bowser E.K., Farnham K., Mangus M. (2021). Effect of CFTR Modulators on Anthropometric Parameters in Individuals with Cystic Fibrosis: An Evidence Analysis Center Systematic Review. J Acad Nutr Diet.

[30] Khare S., Harindhanavudhi T., Wang Q., Espinosa R., Griffin T., Downs E. (2020). Factors Associated with Weight Gain and Improvement in FEV1 in People with Cystic Fibrosis on Elexecaftor-Tezacaftor-Ivacaftor. Pediatr Pulmonol.

[31] Stallings V.A., Sainath N., Oberle M., Bertolaso C., Schall J.I. (2019). Energy Balance and Mechanisms of Weight Gain with Ivacaftor Treatment of Cystic Fibrosis Gating Mutations. J Pediatr.

[32] Borowitz D., Lubarsky B., Wilschanski M., Munck A., Gelfond D., Bodewes F. (2016). Nutritional Status Improved in Cystic Fibrosis Patients with the G551D Mutation After Treatment with Ivacaftor. Dig Dis Sci.

[33] Fryar CD, Carroll MD, Afful J. Prevalence of Overweight, Obesity, and Severe Obesigty Among Adults Aged 20 and Over: United States 1960-1962 through 2017-2018. 2020. https://doi.org/10.1001/jama.2020.14590.

[34] Fryar CD, Carroll MD, Afflul J. Prevalence of overweight, obesity, and severe obesity among children and adolescents aged 2-19 years: United States, 1963-1965 through 2017-2018. vol. NCHS E-Hea. 2020. https://doi.org/10.1001/jama.2020.14590.

[35] Archer E., Lavie C.J., Hill J.O. (2018). The Contributions of ‘Diet’ ‘Genes’ and Physical Activity to the Etiology of Obesity: Contrary Evidence and Consilience. Prog Cardiovasc Dis.

[36] Stallings V.A., Stark L.J., Robinson K.A., Feranchak A.P., Quinton H. (2008). Evidence-Based Practice Recommendations for Nutrition-Related Management of Children and Adults with Cystic Fibrosis and Pancreatic Insufficiency: Results of a Systematic Review. J Am Diet Assoc.

[37] Melo L.C., da Silva M.A.M., Calles A.C.do.N. (2014). Obesity and lung function: a systematic review. Einstein (Sao Paulo).

[38] Alvarez J.A., Ziegler T.R., Millson E.C., Stecenko A.A. (2016). Body composition and lung function in cystic fibrosis and their association with adiposity and normal-weight obesity. Nutrition.

[39] Ramos R.T.T., Salles C., Daltro C.H.D.C., Santana M.A., Gregório P.B., Acosta A.X. (2011). Sleep architecture and polysomnographic respiratory profile of children and adolescents with cystic fibrosis. J Pediatr (Rio J).

[40] Perin C., Fagondes S.C., Casarotto F.C., Pinotti Antônio.F.F., Menna Barreto Sérgio.S., Dalcin P.de.T.R. (2012). Sleep findings and predictors of sleep desaturation in adult cystic fibrosis patients. Sleep Breath.

[41] Shakkottai A, O’Brien LM, Nasr SZ, Chervin RD. Sleep disturbances and their impact in pediatric cystic fibrosis. Sleep Med Rev 2018;42:100–10. https://doi.org/10.1016/j.smrv.2018.07.002. 10.1016/j.smrv.2018.07.002 PMC6221988 30093360

[42] Orens J.B., Estenne M., Arcasoy S., Conte J.V., Corris P., Egan J.J. (2006). International Guidelines for the Selection of Lung Transplant Candidates: 2006 Update-A Consensus Report From the Pulmonary Scientific Council of the International Society for Heart and Lung Transplantation. J Hear Lung Transplant.

[43] Granados A., Chan C.L., Ode K.L., Moheet A., Moran A., Holl R. (2019). Cystic fibrosis related diabetes: Pathophysiology, screening and diagnosis. J Cyst Fibros.

[44] Arrigo T., Cucinotta D., Nibali S.C., Cesare E.D., Benedetto A.D., Magazzù G. (1993). Longitudinal evaluation of glucose tolerance and insulin secretion in non-diabetic children and adolescents with cystic fibrosis: results of a two-year follow-up. Acta Pediatr.

[45] Colomba J., Boudreau V., Lehoux-Dubois C., Desjardins K., Coriati A., Tremblay F. (2019). The main mechanism associated with progression of glucose intolerance in older patients with cystic fibrosis is insulin resistance and not reduced insulin secretion capacity. J Cyst Fibros.

[46] Moran A., Brunzell C., Cohen R.C., Katz M., Marshall B.C., Onady G. (2010). Clinical care guidelines for cystic fibrosis-related diabetes: A position statement of the American Diabetes Association and a clinical practice guideline of the Cystic Fibrosis Foundation, endorsed by the Pediatric Endocrine Society. Diabetes Care.

[47] Ayoub F., Trillo-Alvarez C., Morelli G., Lascano J. (2018). Risk factors for hepatic steatosis in adults with cystic fibrosis: Similarities to non-alcoholic fatty liver disease. World J Hepatol.

[48] Jensen M.D., Ryan D.H., Apovian C.M., Ard J.D., Comuzzie A.G., Donato K.A. (2014). 2013 AHA/ACC/TOS Guideline for the Management of Overweight and Obesity in Adults. Circulation.

[49] Apovian C.M., Aronne L.J., Bessesen D.H., McDonnell M.E., Murad M.H., Pagotto U. (2015). Pharmacological management of obesity: An endocrine society clinical practice guideline. J Clin Endocrinol Metab.

[50] Saxby N, Kench A, King S, Crowder T, van der Haak N. Nutrition Guidelines for Cystic Fibrosis 2017. 10.1016/j.jcf.2019.05.007 31175004

[51] McDonald C.M., Bowser E.K., Farnham K., Alvarez J.A., Padula L., Rozga M. (2021). Dietary Macronutrient Distribution and Nutrition Outcomes in Persons with Cystic Fibrosis: An Evidence Analysis Center Systematic Review. J Acad Nutr Diet.

[52] Butler P.C., Dry S., Elashoff R. (2010). GLP-1 – Based Therapy for Diabetes : What You Do Not Know Can Hurt You. Diabetes Care.

[53] Bray G.A., Frühbeck G., Ryan D.H., Wilding J.P.H. (2016). Management of obesity. Lancet.

[54] Arterburn DE, Courcoulas AP. Bariatric surgery for obesity and metabolic conditions in adults. BMJ 2014;349:2012–3. https://doi.org/10.1136/bmj.g3961. 10.1136/bmj.g3961 PMC4707708 25164369

[55] Turck D., Braegger C.P., Colombo C., Declercq D., Morton A., Pancheva R. (2016). ESPEN-ESPGHAN-ECFS guidelines on nutrition care for infants, children, and adults with cystic fibrosis. Clin Nutr.

[56] Gadde K.M., Allison D.B., Ryan D.H., Peterson C.A., Troupin B., Schwiers M.L. (2011). Effects of low-dose, controlled-release, phentermine plus topiramate combination on weight and associated comorbidities in overweight and obese adults (CONQUER): A randomised, placebo-controlled, phase 3 trial. Lancet.

[57] LeBlanc E, O’Connor E, Whitlock E, Patnode C, Kapka T. Review Annals of Internal Medicine Effectiveness of Primary Care – Relevant Treatments for Obesity in. Ann Intern Med 2011;155:434–47. 10.7326/0003-4819-155-7-201110040-00006 21969342

[58] Greenway F.L., Fujioka K., Plodkowski R.A., Mudaliar S., Guttadauria M., Erickson J. (2010). Effect of naltrexone plus bupropion on weight loss in overweight and obese adults (COR-I): A multicentre, randomised, double-blind, placebo-controlled, phase 3 trial. Lancet.

[59] Wilding J.P.H., Batterham R.L., Calanna S., Davies M., Van Gaal L.F., Lingvay I. (2021). Once-Weekly Semaglutide in Adults with Overweight or Obesity. N Engl J Med.

[60] Pi-Sunyer X., Astrup A., Fujioka K., Greenway F., Halpern A., Krempf M. (2015). A Randomized, Controlled Trial of 3.0 mg of Liraglutide in Weight Management. N Engl J Med.

